# Recognition of Thiols in Living Cells and Zebrafish Using an Imidazo[1,5-α]pyridine-Derivative Indicator

**DOI:** 10.3390/molecules24183328

**Published:** 2019-09-12

**Authors:** Song Chen, Peng Hou, Jingwen Sun, Haijun Wang, Lei Liu

**Affiliations:** College of Pharmacy, Qiqihar Medical University, Qiqihar 161006, China; penghou@csu.edu.cn (P.H.); sewage2010@163.com (J.S.); wanghaijun@qmu.edu.cn (H.W.); liuleiyx@qmu.edu.cn (L.L.)

**Keywords:** large Stokes shift, zebrafish, thiols, cell imaging, imidazo[1,5-α]pyridine

## Abstract

A new cyan fluorescent probe, **MIPY-DNBS**, using an imidazo[1,5-α]pyridine derivative as the fluorophore and 2,4-dinitrobenzensufonate as the recognition site for the selective detection of thiols (Cys, GSH, and Hcy), was designed and synthesized. Probe **MIPY-DNBS** exhibited a 172 nm Stokes shift, a fast response time (400 s), low cytotoxicity, low detection limits (12.7 nM for Cys), and excellent selectively in the detection of thiols. In addition, **MIPY-DNBS** was successfully applied to imaging thiols in living MCF-7 cells and zebrafish.

## 1. Introduction

Among diverse mercapto biomolecules, cysteine (Cys), homocysteine (Hcy), and glutathione (GSH) as intracellular small-molecule thiols have attracted more interest due to their playing vital roles in maintaining biological systems [1,2,3]. Cys and Hcy are essential biological molecules and involved in cellular growth. GSH is the most abundant intracellular non-proteinogenic thiol and serves as a redox regulator [4,5,6]. However, the abnormal fluctuation in levels of Cys, Hcy, or GSH will be relative with various health problems. The alteration of Cys levels is implicated in edema, retarded growth, liver damage, and skin lesions [7,8,9]. The rise in Hcy content is a dangerous sign of cardiovascular and Alzheimer’s diseases [10,11]. The insufficiency of GSH can cause diseases linked with oxidative stress, such as neural-tube defects, osteoporosis, and cancer [12,13]. Thus, it is of great importance to develop an efficient method for the recognition and quantification of thiols to better research their biological and pathological roles.

Various analytical methods, including chromatographic, electrochemical, and titrimetric, were developed for detecting thiols [14,15,16,17]. Given the advantages of the real-time and nondestructive detection on the living biosamples, fluorescence sensors have attracted considerable attention as effective molecular-recognition tools in vivo [18,19,20]. While numerous fluorescent probes were designed specifically for thiols assay [21,22,23,24,25,26,27,28,29,30,31], most of them respond toward thiols with small Stokes shifts (Table S1). It’s known that fluorescence probes with small Stokes shifts are usually associated with a decrease in sensitivity that results from the overlap of excitation and emission spectra. As a consequence, it is of great significance to explore highly selective fluorescent probes with large stokes shifts for sensitively tracing biothiols in vivo.

The imidazo[1,5-α]pyridine ring system and its derivatives possess desirable photophysical properties, such as good photostabilities, emission in the cyan region, and large Stokes shifts [32]. In this work, we present a new imidazo[1,5-α]pyridine-based fluorescence probe, **MIPY-DNBS**, for the detection of thiols. The imidazo[1,5-α]pyridine platform, **MIPY-OH,** was introduced as the fluorophore core, and the 2,4-dinitrobenzensufonate moiety acted as the recognition site and quencher. Owing to the photoinduced electron transfer (PET) process, **MIPY-DNBS** itself showed weak fluorescence. Upon the addition of thiols (Cys, Hcy, and GSH), the PET process of **MIPY-DNBS** was blocked, and the ESIPT (excited state intramolecular proton transfer) of that was restored, accompanied by a dramatic fluorescence response. Moreover, **MIPY-DNBS** exhibited a large Stokes shift in the detection of thiols, which could help to reduce any possible self-quenching and self-fluorescence [33,34,35]. Importantly, **MIPY-DNBS** was successfully applied to monitoring thiols in living MCF-7 cells and zebrafish.

## 2. Results and Discussion

### 2.1. Design and Spectroscopic Studies

**Scheme 1:**
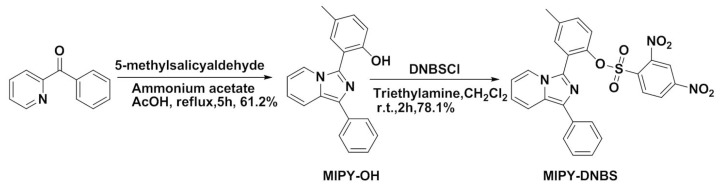
Synthetic route of MIPY-DNBS.

**Figure 1:**
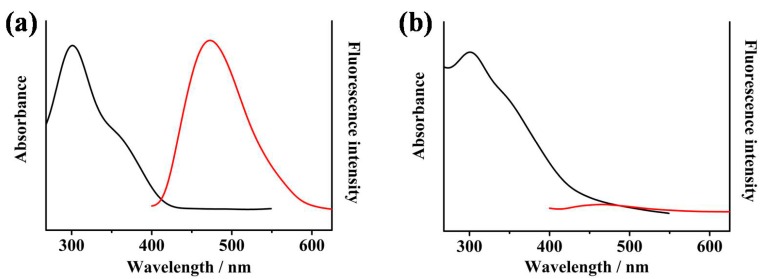
The emission spectra (red line) and absorption (black line) of **MIPY-OH** (**a**) and **MIPY-DNBS** (**b**) in pH 7.4 phosphate-buffered saline (PBS) buffer (50.0 mM, containing 20% DMSO).

For the design of a thiols-fluorescence chemosensor based on the PET and ESIPT dual-quenching strategy, we utilized the imidazo[1,5-α]pyridine derivative **MIPY-OH** as the electron donor and 2,4-dinitrobenzensufonate as the electron acceptor. The quenching fluorescence was in probe **MIPY-DNBS** via the PET and ESIPT process. In the recognition process, the 2,4-dinitrobenzensufonate ether group could be deprotected by thiols to lead a significant cyan fluorescence with the inhibition of PET and the recovery of ESIPT. The synthetic route of **MIPY-DNBS** is shown in Scheme 1. The structure of **MIPY-DNBS** in this strategy was verified by NMR and mass spectra. The UV-VIS absorbance and fluorescence behaviors of **MIPY-DNBS** and **MIPY-OH** were evaluated in 7.4 phosphate-buffered saline (PBS) buffers (50.0 mM, containing 20% DMSO). As exhibited in Figure 1, **MIPY-OH** (10 μM) showed a maximum absorption at 301 nm and a notable fluorescence at 473 nm, which displayed a 172 nm Stokes shift. However, **MIPY-DNBS** (10 μM) was essentially nonfluorescent in the same emission range. The distinction of fluorescence behaviors demonstrated that **MIPY-DNBS** was able to be an efficient fluorescent switch probe for detecting thiols.

### 2.2. Sensing Properties of MIPY-DNBS to Thiols

**Figure 2:**
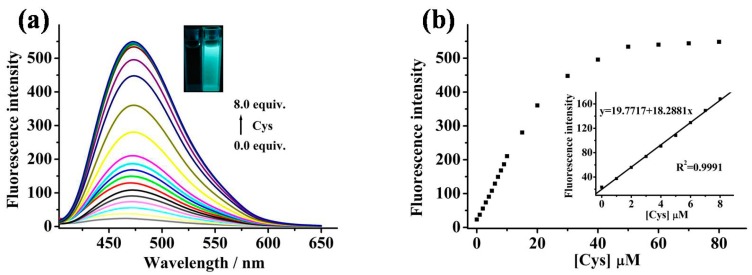
(**a**) Fluorescence response of **MIPY-DNBS** (10 μM) treated with the addition of Cys (0–80 µM) in PBS buffer (50.0 mM, containing 20% DMSO). Inset: Fluorescence images of **MIPY-DNBS** solution in the absence (left) and presence (right) of Cys under a 365 nm UV lamp. (**b**) Fluorescence intensity of **MIPY-DNBS** (10 μM) at 473 nm as a function of Cys concentration in PBS buffer. Inset: The linear relationship of concentration-dependent fluorescence-intensity changes.

**Scheme 2:**
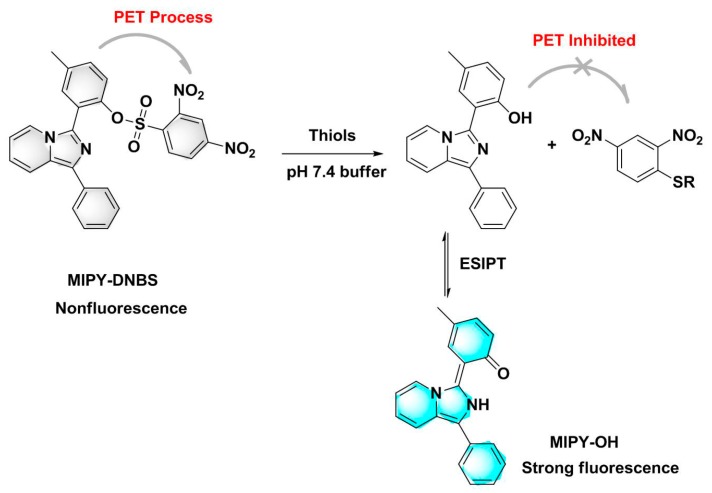
A proposed mechanism for the detection of thiols.

To demonstrate the sensing behavior of **MIPY-DNBS** for detecting thiols quantitatively, the fluorescence response of the **MIPY-DNBS** solution treated with a series of different concentrations of thiols (Cys, Hcy, and GSH) was evaluated. When the increasing doses of Cys (0–80 μM) were added to the **MIPY-DNBS** (10 μM) solution, the fluorescence enhancement at 473 nm was within detection limits (Figure 2a), indicating that **MIPY-OH** generated from the reaction of **MIPY-DNBS** and Cys (Scheme 2). The strategy in this work was further verified by the HRMS spectra (Figure S10). As expected, the mixture of **MIPY-DNBS** with Cys (*m*/*z* = 301.1349) and **MIPY-OH** (cal. 301.1341) almost had the same molecular weight. What’s more, the fluorescence intensity had a good linear relationship (y = 19.7717 + 18.2881x, R^2^ = 0.9991), with the Cys over the concentration range from 0 to 8 μM. Based on S/N = 3, 12.7 nM was obtained for the detection limit of **MIPY-DNBS** toward Cys (Figure 2b). Furthermore, the similar fluorescence-change trends were shown, in which the fluorescence intensity of **MIPY-DNBS** was linearly correlation dependent on Hcy and GSH (Figures S1–S4). The detection limits were determined to be 20.4 and 53.1 nM for GSH and Hcy, respectively. The above results suggested probe **MIPY-DNBS** can be quantitatively employed to monitor thiols in an aqueous solution with ultrahigh sensitivity.

### 2.3. Specificity Evaluation

**Figure 3:**
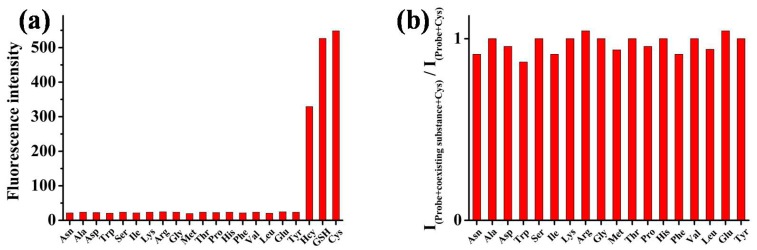
(**a**) Fluorescence response of **MIPY-DNBS** (10.0 μM) to common amino acids. Data shown are for 160 μM of Asn, Ala, Asp, Trp, Ser, Ile, Lys, Arg, Gly, Met, Thr, Pro, His, Phe, Val, Leu, Glu, Tyr and 80 μM of Cys, Hcy, and GSH) at 473 nm in PBS buffer (50.0 mM, containing 20% DMSO). (**b**) Fluorescence response of **MIPY-DNBS** (10.0 μM) to Cys (80 μM) with various interferences, including 160 μM for Asn, Ala, Asp, Trp, Ser, Ile, Lys, Arg, Gly, Met, Thr, Pro, His, Phe, Val, Leu, Glu, and Tyr in PBS buffer (50.0 mM, containing 20% DMSO).

The selectivity and competition measurements were carried out. Compared with the common amino acids (Figure 3a) and other analytes (Figure S5) in the biological system, **MIPY-DNBS** (10 μM) exhibited a significant enhancement to thiols over other analytes (160 μM) (Asn, Ala, Asp, Trp, Ser, Ile, Lys, Arg, Gly, Met, Thr, Pro, His, Phe, Val, Leu, Glu, Tyr, Na^+^, Mn^2+^, K^+^, Fe^3+^, Zn^2+^, Mg^2+^, Ca^2+^, H_2_O_2_, NADH, and citric acid). Moreover, the competitive data showed an obvious response for **MIPY-DNBS** (10 μM) to recognize Cys (80 μM), with the addition of the representative interfering substance (160 μM) (Figure 3b, Figure S6). The data of probe **MIPY-DNBS** toward Cys accompanied with other analytes corroborated that **MIPY-DNBS** possessed satisfactory selectivity to sense thiols in complicated sample conditions.

### 2.4. pH Stability on Thiols Studies

**Figure 4:**
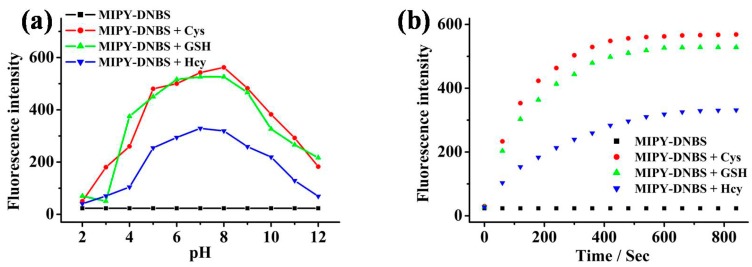
(**a**) Fluorescence response at 473 nm of **MIPY-DNBS** (10.0 μM) in the absence and presence of Cys (80 μM) at different pH values. (**b**) Time-dependent fluorescence intensity of **MIPY-DNBS** (10 μM) at 473 nm before and after Cys (80 μM) addition in PBS buffer (50.0 mM, containing 20% DMSO).

In biological applications, a favorable pH is essential for the reaction between **MIPY-DNBS** and thiols. The pH effect of the fluorescence intensity for **MIPY-DNBS** (10 μM) in the absence and presence of thiols (80 μM) was investigated (Figure 4a). It was observed that the free **MIPY-DNBS** was stable and had a negligible fluorescence change in the pH range of 2–12. However, when thiols (Cys, Hcy, and GSH) were added to the solution of **MIPY-DNBS**, the three-group fluorescence intensity of **MIPY-DNBS** reacted with Cys, Hcy, and GSH, and it represented a remarkable increase in fluorescence and reached the maximum in the pH range from 6 to 8. It is implied that **MIPY-DNBS** is able to detect thiols in the physiological pH region.

### 2.5. Reaction Time on Sensing Thiols

To elaborate the real-time detection capability of **MIPY-DNBS** against thiols, another important parameter, the time course on fluorescence intensity of the responses of **MIPY-DNBS** (10 μM) to Cys, Hcy, and GSH (80 μM) were investigated at the physiological pH. As shown in Figure 4b, the fluorescence of free **MIPY-DNBS** remained silent during the measurement, and it became almost constant. In contrast, when **MIPY-DNBS** was treated with Cys, Hcy, and GSH, the fluorescence intensity (473 nm) showed a rapid increase with time, and it reached a plateau around 400 s. It is suggested that **MIPY-DNBS** is sensitive for rapid monitoring thiols in an aqueous medium.

### 2.6. Fluorescence Imaging in Living MCF-7 Cells

**Figure 5:**
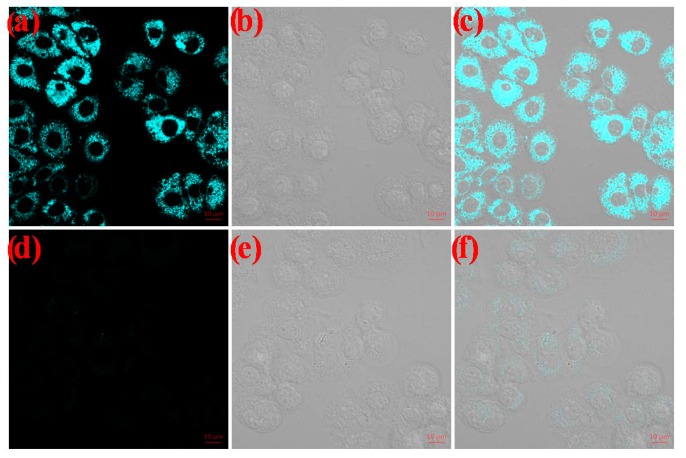
Confocal fluorescence images of MCF-7 cells. Conditions: MCF-7 cells stained with 10 μM **MIPY-DNBS** for 30 min (**a**–**c**); NEM-pretreated MCF-7 cells incubated with 10 μM **MIPY-DNBS** for 30 min (**d**–**f**). Scale bar: 10 μm. (**a**,**d**) Fluorescence field images; (**b**,**e**) bright field images; (**c**,**f**) merge images of bright field and fluorescence field. Excitation wavelength: 405 nm. Emissions were collected at 460–510 nm for cyan channel.

To evaluate the practical utilities of **MIPY-DNBS** in living cells, we first measured the cytotoxicity of **MIPY-DNBS** toward MCF-7 cells by the standard MTT assays. The cellular viability results exhibited that **MIPY-DNBS** is safe and has low toxicity in cellular studies, as greater than 93% of cells survived at the concentration of a 10 μM probe for as long as 24 h (Figure S7). When the MCF-7 cells were treated with **MIPY-DNBS** (10 μM) for 30 min, a dramatic cyan intracellular fluorescence signal was observed (Figure 5), which indicated that **MIPY-DNBS** had good cell permeability and reacted with endogenous thiols (the reaction product may be accumulating in lipid droplets or lysosomes). As a control, cells were pre-cultured with a thiols-trapping reagent (1.0 mM N-ethylmaleimide, NEM) for 30 min, and then cultured with **MIPY-DNBS** (10 μM) for 30 min. No florescence signal was detected intracellularly, since endogenous thiols were inhibited. Based on the results, we deduced that **MIPY-DNBS** was potentially capable of imaging thiols for biological application.

### 2.7. Fluorescence Imaging in Zebrafish

**Figure 6:**
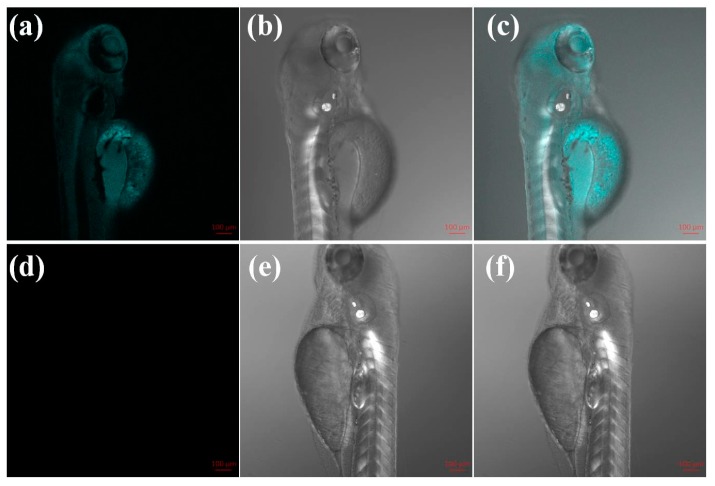
Imaging experiments for detection of thiols in zebrafish. (**a**–**c**) Zebrafish incubated with probe **MIPY-DNBS** (10.0 µM) for 30 min. (**d**–**f**) Zebrafish pretreated with 1 mM NEM for 30 min, then incubated with probe **MIPY-DNBS** (10.0 µM) for another 30 min. Scale bar: 100 μm. (**a**,**d**) Fluorescence field images; (**b**,**e**) bright field images; (**c**,**f**) merge images of bright field and fluorescence field.

In view of the favorable optical property of **MIPY-DNBS** for in vitro and cellular-imaging studies, further experiments were carried out to visualize thiols in living zebrafish. As seen in Figure 6, there was stronger fluorescence found as a result of the fact that **MIPY-DNBS** (10 μM) bound thiols in zebrafish. While, in the case of NEM-pretreated zebrafish that were incubated in **MIPY-DNBS** (10 μM), no fluorescence emission was observed, which was consistent with cell-imaging studies. These results confirmed that **MIPY-DNBS** was capable of imaging thiols in living animals.

## 3. Materials and Methods

### 3.1. Instruments and Chemicals

The absorption spectra and emission spectra were recorded using a UV-Vis 2450 instrument (Shimadzu, Kyoto, Japan) and an RF5301PC fluorescence fluorometer (Shimadzu, Kyoto, Japan). The emission spectra were performed on a set 5.0 nm for excitation and emission slit widths. The fluorescence imaging in vivo was obtained by a Zeiss LSM710 microscope (Jena, Germany). The pH experiment was adjusted by a PHS-3C pH meter (Leici, Shanghai, China). A Waters ^®^ Xevo G2-S QTof™ mass spectrometer was used for mass spectra (Waters^®^, Manchester, UK), and a BRUKER 600 spectrometer was used for NMR spectra (Rheinstetten, Germany). No further purification was operated for all reagents before work. The thin-layer chromatography (TLC) plates and silica gel (mesh 200–300) were purchased from Qingdao Chemical (Qingdao, China).

### 3.2. Spectroscopic Methods

The stock solution of **MIPY-DNBS** was prepared in DMSO for 1.0 mM. The stock solution of analytes (Asn, Ala, Asp, Trp, Ser, Ile, Lys, Arg, Gly, Met, Thr, Pro, His, Phe, Val, Leu, Glu, Tyr, Cys, Hcy, GSH, Na^+^, Mn^2+^, K^+^, Fe^3+^, Zn^2+^, Mg^2+^, Ca^2+^, H_2_O_2_, NADH, citric acid) in double-distilled water was prepared and diluted by 7.4 PBS (50.0 mM). For the measurement solution, 0.03 mL of **MIPY-DNBS** stock solution was placed in a 3 mL volume quartz cuvette and mixed with the appropriated analytes solution. After the reaction solution was shaken well for 400 s at room temperature, measurements were recorded by UV-VIS absorbance and fluorescence spectrum.

### 3.3. Synthesis of Probe MIPY-DNBS

To a solution of **MIPY-OH** (105.1 mg, 0.35 mmol), 2,4-dinitrobenzenesulfonyl chloride (125.3 mg, 0.47 mmol), and CH_2_Cl_2_ (15 mL) was added triethyl amine (47.6 mg, 0.47 mmol). After stirring at room temperature under argon atmosphere for 2 h, the resulting compound was further purified by chromatography on silica gel, using CH_2_Cl_2_ as an eluent to yield the probe **MIPY-DNBS** (78.1%). ^1^H-NMR (600 MHz, DMSO) δ 8.49 (d, *J* = 2.3 Hz, 1H), 8.16 (dd, *J* = 8.7, 2.3 Hz, 1H), 7.83 (dd, *J* = 9.5, 8.5 Hz, 2H), 7.70 (d, *J* = 7.2 Hz, 2H), 7.59 (d, *J* = 8.7 Hz, 1H), 7.55 (d, *J* = 1.0 Hz, 2H), 7.50 (s, 1H), 7.39 (t, *J* = 7.7 Hz, 2H), 7.27 (t, *J* = 7.3 Hz, 1H), 6.98–6.89 (m, 1H), 6.81–6.72 (m, 1H), 2.44 (s, 3H). HRMS (EI) *m*/*z* calcd for [C_26_H_18_N_4_O_7_S + H]^+^: 531.0974, Found: 531.0971.

### 3.4. Cells Culture and Fluorescence Imaging

The MCF-7 cells were bred in a Dulbecco minimum essential medium (DMEM) nutrient fluid, which was modified with Eagle’s medium and supplemented with 10% fetal bovine serum (FBS) under an atmosphere of 37 °C and 5% CO_2_ gas. For the imaging, the cells were incubated in glass-bottom dishes for 24 h, and then they were then treated with 10 μM **MIPY-DNBS** for 30 min at 37 °C. After removing the residual solution with PBS, the cell-fluorescence imaging was recorded with a confocal microscope. For the control group, the cells were pretreated with 1 mM of N-ethylmaleimide (NEM) for 30 min prior to the 10 μM of **MIPY-DNBS**, loaded for 30 min at 37 °C, and handled in the same washing way. All cell imaging was measured using a Zeiss LSM710 laser confocal microscope reader.

### 3.5. Fluorescence Imaging in Zebrafish

The 3-day-old zebrafish were placed in microplates and trained with a 10 μM **MIPY-DNBS** solution for 30 min. Then, imaging was performed, and they were washed three times with PBS. As a control, zebrafish were pre-seeded with 1 mM NEM and then stained with **MIPY-DNBS** for 30 min for each incubation. Subsequently, the residue was cleared with PBS several times, and the zebrafish imaging was measured. The zebrafish experimental operations were conducted according to the National Institutes of Health guide for the use and care of experimental animals and were approved by the Animal Experimentation Ethics Committee of Qiqihar Medical University (2019030607).

## 4. Conclusions

In conclusion, we presented a new cyan fluorescent probe, **MIPY-DNBS**, using an imidazo[1,5-α]pyridine derivative as the fluorophore and 2,4-dinitrobenzensufonate as the recognition site for the selective detection of thiols (Cys, GSH, and Hcy). In the detection of thiols, **MIPY-DNBS** displayed a fast response time (400 s), low cytotoxicity, a 172 nm Stokes shift, excellent selectively, and low detection limits (12.7 nM for Cys). More importantly, **MIPY-DNBS** was applied for sensing thiols in living MCF-7 cells and zebrafish successfully.

## Supplementary Materials

The following are available online at https://www.mdpi.com/1420-3049/24/18/3328/s1: Figure S1 Fluorescence spectra changes of MIPY-DNBS (10 μM) upon the addition of GSH (0–80 μM) in pH 7.4 PBS buffer. Figure S2 Fluorescence intensity of MIPY-DNBS (10 μM) at 473 nm as a function of GSH concentration (0–80 μM) in pH 7.4 PBS buffer. Inset: the linear relationship between fluorescence intensity and GSH at low concentrations. Figure S3 Fluorescence spectra changes of MIPY-DNBS (10 μM) upon the addition of Hcy (0–80 μM) in pH 7.4 PBS buffer. Figure S4 Fluorescence intensity of MIPY-DNBS (10 μM) at 473 nm as a function of Hcy concentration (0–80 μM) in pH 7.4 PBS buffer. Inset: the linear relationship between fluorescence intensity and Hcy at low concentrations. Figure S5 The fluorescence intensity at 473 nm of MIPY-DNBS (10 μM) upon the addition of the various analytes. Figure S6 The fluorescence intensity at 473 nm of MIPY-DNBS (10 μM) to Cys (80 μM) with the competition analytes in pH 7.4 PBS buffer. Figure S7 Cytotoxicity assay of MIPY-DNBS at different concentrations for MCF-7 cells. Figure S8 ^1^H-NMR spectrum of MIPY-DNBS in DMSO-*d_6_*. Figure S9 Mass spectrum of MIPY-DNBS. Figure S10 Mass spectrum of the reaction product of MIPY-DNBS with Cys. Table S1 Fluorescent probes for biothiols.
