# Supplementary material for: Recognition of Thiols in Living Cells and Zebrafish Using an Imidazo[1,5-α]pyridine-Derivative Indicator
Source: Molecules. 2019 Sep 12;24(18):3328. doi: 10.3390/molecules24183328 (PMC6767008; doi:10.3390/molecules24183328)
Supplement: Supplementary file 1 [file molecules-24-03328-s001.pdf]

Supporting information for

Recognition of thiols in living cells and zebrafish using an imidazo[1,5-α]pyridine derivative indicator

Song Chen *, Peng Hou, Jingwen Sun, Haijun Wang, Lei Liu

College of Pharmacy, Qiqihar Medical University, 333 Bukui Street, Qiqihar, Heilongjiang Province, P. R. China, 161006.

* Corresponding author.

E-mail address: songchen@csu.edu.cn

Table of contents

Page

Figures S1-2 .... **S2**

Figures S3-4 .... **S3**

Figures S5-6 .... **S4**

Figures S7-8 .... **S5**

Figures S9-10 .... **S6**

TableS1.. **S7**

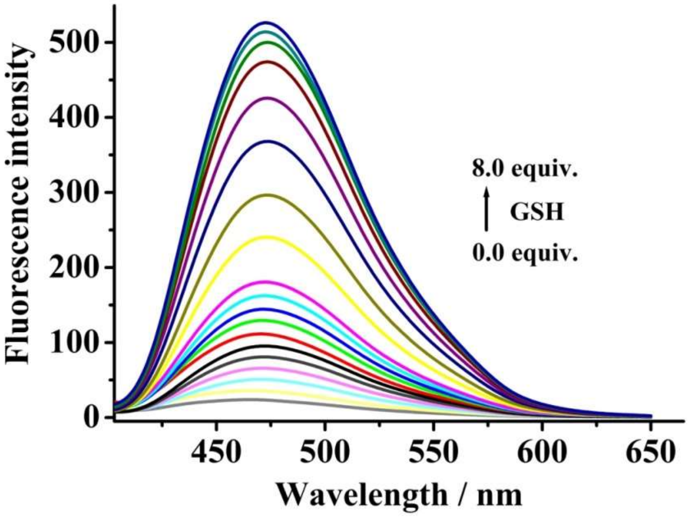

Figure S1 Fluorescence spectra changes of **MIPY-DNBS** (10 μM) upon the addition of GSH (0 –80 μM) in pH 7.4 PBS buffer.

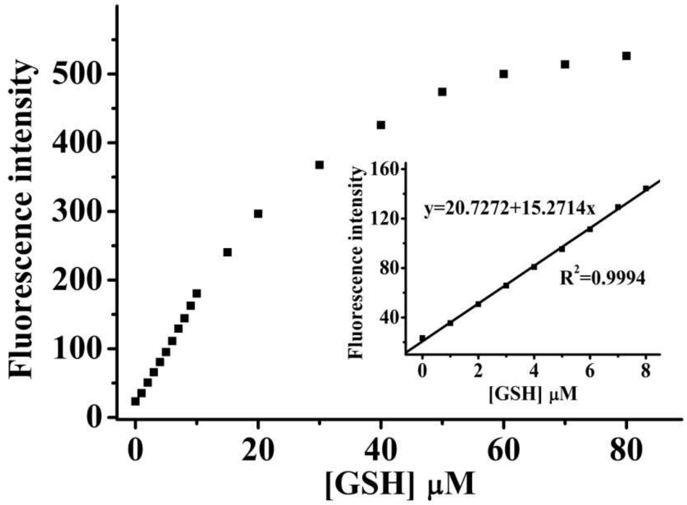

Figure S2 Fluorescence intensity of **MIPY-DNBS** (10 μM) at 473 nm as a function of GSH concentration (0 –80 μM) in pH 7.4 PBS buffer. Inset: the linear relationship between fluorescence intensity and GSH at low concentrations.

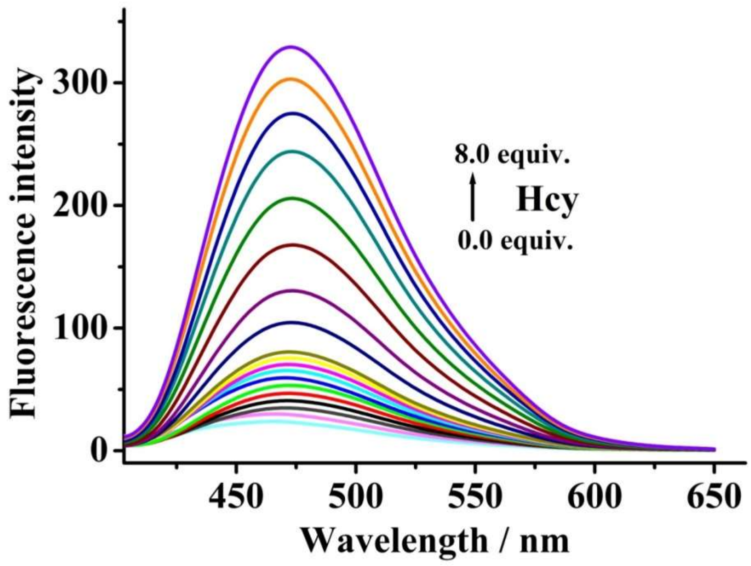

Figure S3 Fluorescence spectra changes of **MIPY-DNBS** (10 μM) upon the addition of Hcy (0 –80 μM) in pH 7.4 PBS buffer.

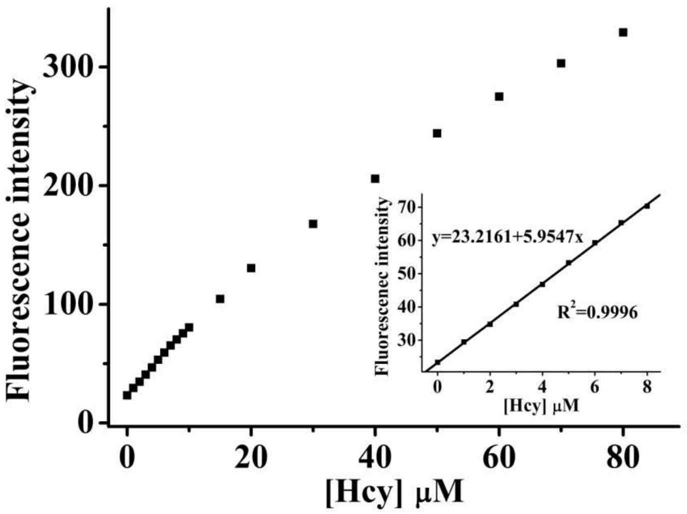

Figure S4 Fluorescence intensity of **MIPY-DNBS** (10 μM) at 473 nm as a function of Hcy concentration (0 –80 μM) in pH 7.4 PBS buffer. Inset: the linear relationship between fluorescence intensity and Hcy at low concentrations.

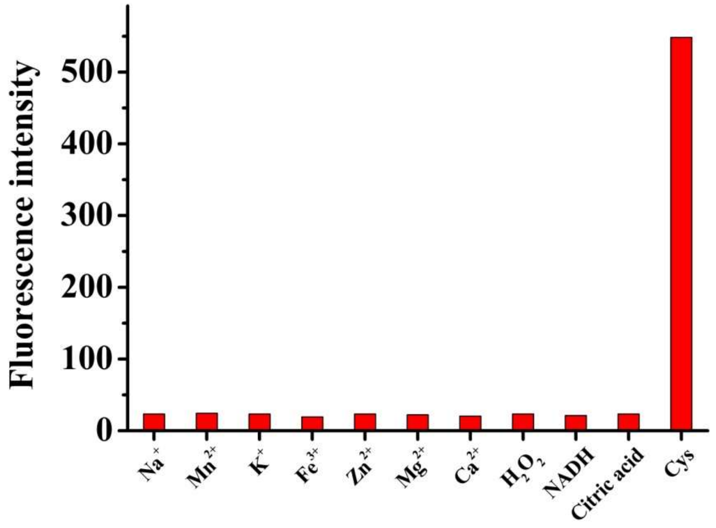

Figure S5 The fluorescence intensity at 473 nm of **MIPY-DNBS** (10 μM) upon the addition of the various analytes.

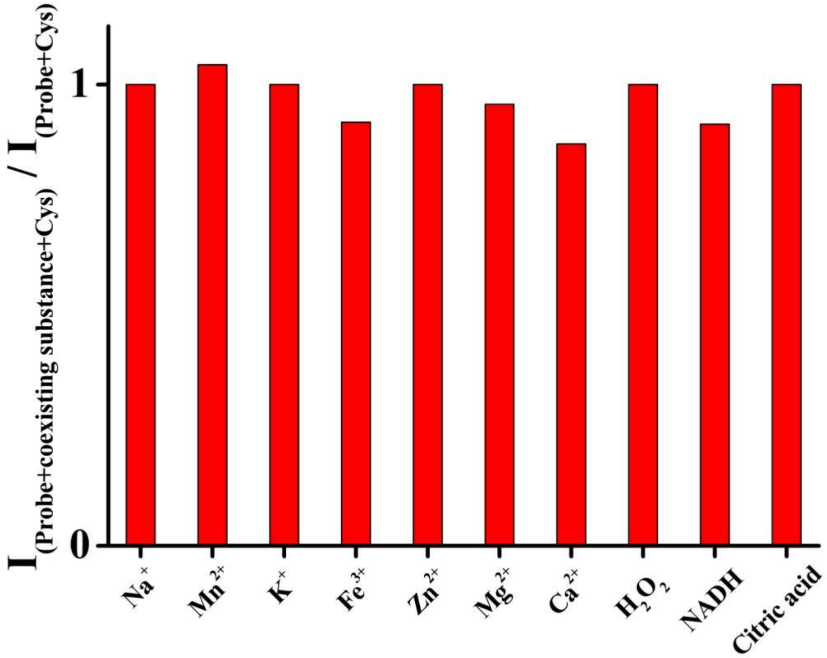

Figure S6 The fluorescence intensity at 473 nm of **MIPY-DNBS** (10 μM) to Cys (80 μM) with the competition analytes in pH 7.4 PBS buffer.

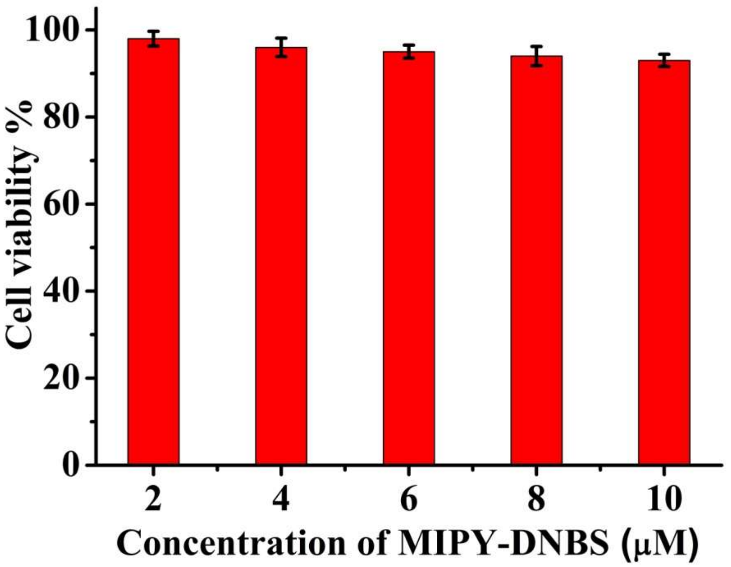

Figure S7 Cytotoxicity assay of **MIPY-DNBS** at different concentrations for MCF-7 cells.

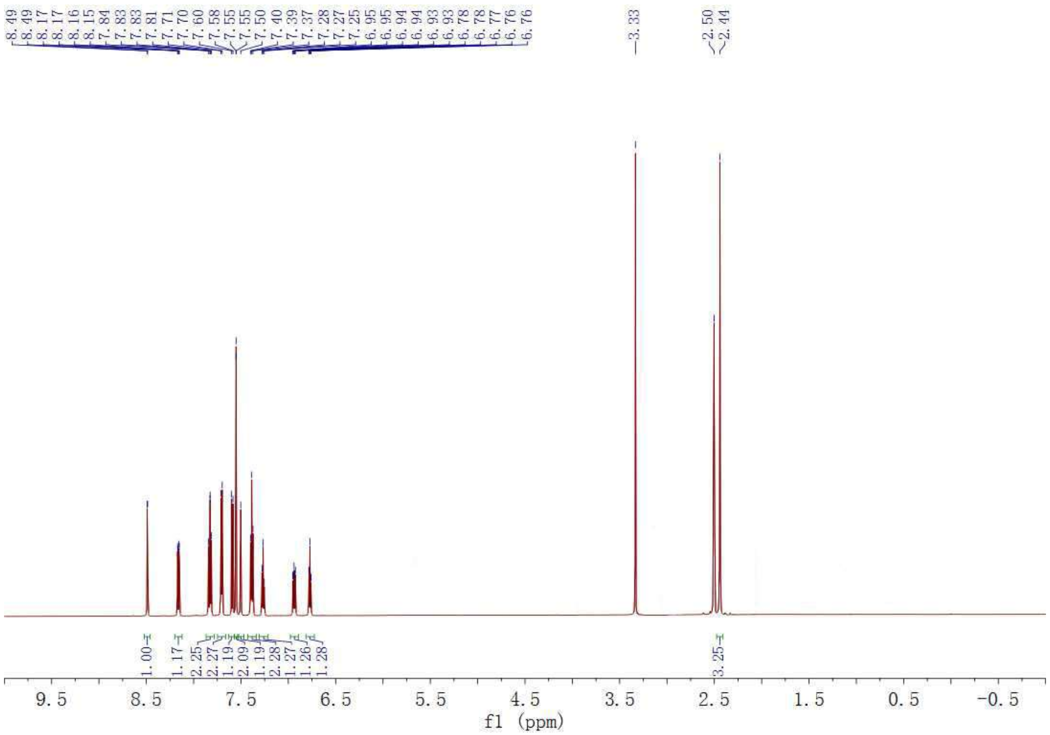

Figure S8 ^{1}H NMR spectrum of **MIPY-DNBS** in DMSO-d_6.

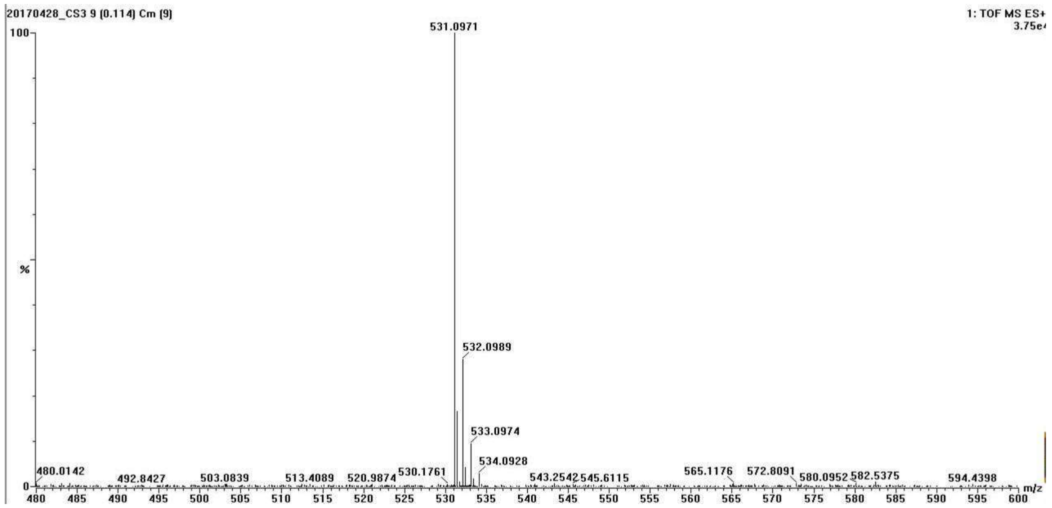

Figure S9 Mass spectrum of **MIPY-DNBS**.

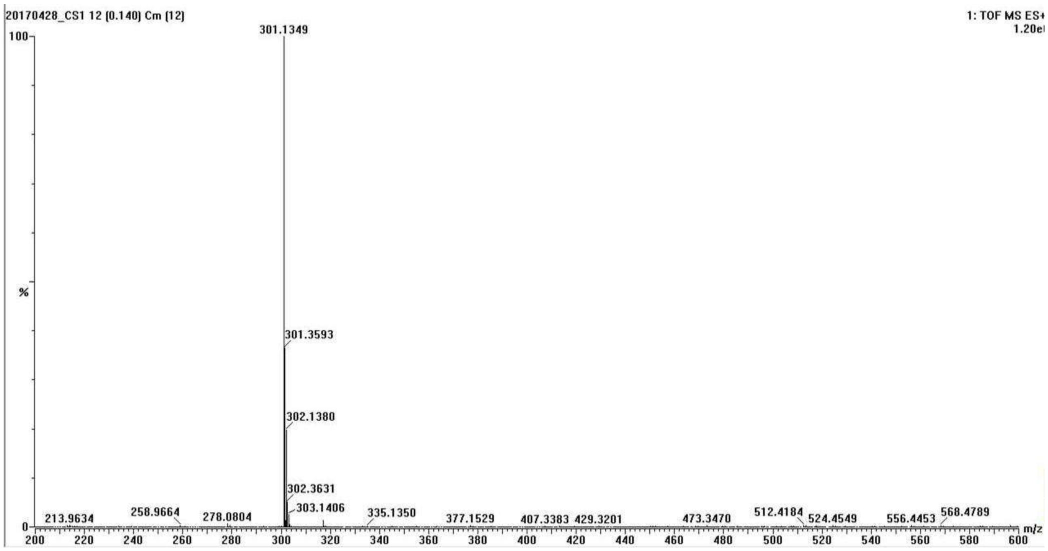

Figure S10 Mass spectrum of the reaction product of M**IPY-DNBS** with Cys.

Table S1 Fluorescent probes for biothiols.

Compound	λex/λem (nm)	Stokes Shift (nm)	LOD	Response Time	Reference
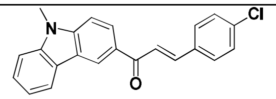	342/470	128	1.770 μM	10 min	Sensor Actuat B-Chem, 2018, 259, 233-240.
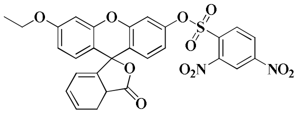	454/521	67	0.16 μM	10 min	Tetrahedron Letters, 2016,57, 2478-2483
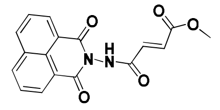	310/394	84	0.2 μM	30 min	Sensor Actuat B-Chem, 2017, 242, 865-871
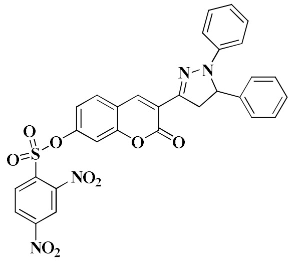	450/540	90	1.5×10^{-8}M	10 min	Sensor Actuat B-Chem, 2016, 223, 274-279.
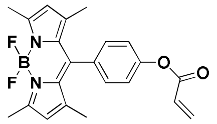	480/517	37	0.05 μM	5 min	Dyes Pigments, 2017, 139, 381-387.
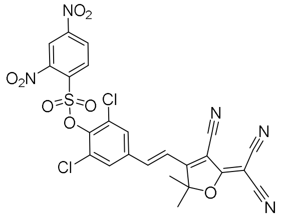	560/625	65	0.45 μM	15 min	Chem. Commun. 2018, 54, 4786-4789
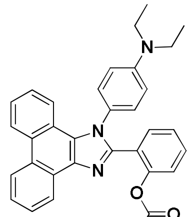	360/465	105	0.64 μM	10 min	Sensor Actuat B-Chem, 2016，233，173-179
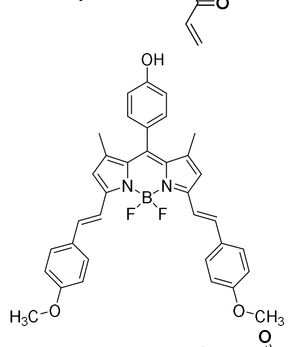	646/656	10	131 nM	90 min	Dyes Pigments, 2018, 152, 85-92
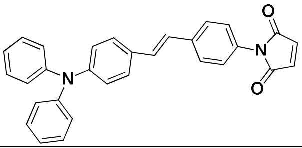	370/480	110	0.13 μM	2.5 min	Dyes Pigments, 2016,128, 209-214

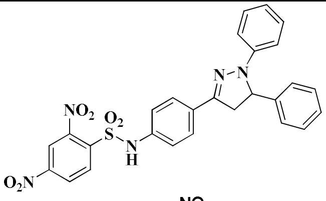	370/464	94	4.11×10^{-7}M	12 h	Analyst, 2013, 138, 7169-7174.
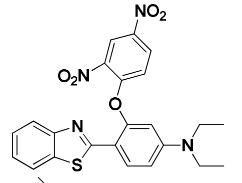	366/423	57	0.084 μM	30 min	Analytical Methods, 2016, 8(38),6832-6839.
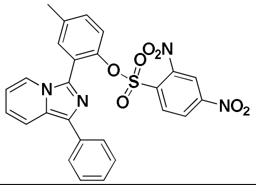	**301/473**	**172**	**12.7 nM**	**400 s**	**This work**
